# Drowning in delays: a patient journey mapping analysis of child drowning care in rural India

**DOI:** 10.1136/bmjopen-2025-103099

**Published:** 2025-08-24

**Authors:** Jagnoor Jagnoor, Medhavi Gupta

**Affiliations:** 1 Injury Division, The George Institute for Global Health, New Delhi, India; 2 Injury Division, The George Institute for Global Health, Sydney, New South Wales, Australia

**Keywords:** Community child health, Community-Based Participatory Research, Risk Factors, PUBLIC HEALTH

## Abstract

**Abstract:**

**Objectives:**

Immediate or urgent and appropriate postevent response to child drowning is crucial to decrease mortality and morbidity. However, these responses are often delayed, especially in low-income contexts. In this study, we use patient journey mapping to identify key delays to care occurring after child drowning incidents in a low-resource, high-risk region.

**Setting:**

The study was conducted in the Sundarbans, a rural region in India facing a high burden of child drowning due to high presence of open water and regular flooding.

**Participants:**

Using purposive sampling, recent child drowning events in the Sundarbans were identified and in-depth interviews were conducted with adults present throughout the rescue and postevent journey to record decisions and delays.

**Design:**

Postdrowning event delays identified through in-depth interviews were mapped against the three-delay framework, encompassing the delays of deciding to seek medical care, transporting the victim to medical facilities and delays in receiving appropriate care at the facility. In addition to the delays identified in the model, we hypothesised that an further delay occurred at rescue, which we called delay zero.

**Results:**

We found evidence for ‘delay zero’ at the time of rescue, which was caused by poor supervision preventing early identification of the event, and unclear water and nighttime both increasing the difficulty of searching for the child. We additionally documented major delays when deciding to attain medical care as bystanders first applied traditional resuscitation methods. Distance to formal medical facilities constituted a barrier, so local bystanders enlisted the help of untrained local doctors before attempting travel.

**Conclusions:**

The results highlighted the critical need to improve child supervision and employ preventative programmes as once drowning events occur, appropriate care was rarely applied in this context. However, postdrowning care could be improved by training bystanders and local informal doctors in cardiopulmonary resuscitation, and by expanding the health system’s geographical reach.

**Strengths and limitations of this study**

- Our study extended the three-delay framework to be applicable to drowning by adding ‘delay zero’—a delay at the time of rescue.
- Our study conducted multiple in-depth interviews to ensure thorough coverage and understanding of postdrowning event journeys.
- Drowning cases were from multiple regions of the Sundarbans to ensure a variety of challenges to postdrowning care were captured.
- The findings of this study may only be generalisable to similar low-income and middle-income contexts to the Sundarbans.
- We did not collect socioeconomic data such as income or occupation, but this may have been insightful to identify challenges related to social factors.

## Introduction

 Rapid and appropriate response to child drowning events is essential to improving survival rates and reducing morbidity from drowning.^1 2^ However, the ability of the health system to respond to drowning events depends on medical capabilities, available resources and information sharing, which are often wanting in low-income contexts.^3^ Best practice recommends that if a child is pulled from the water not breathing, cardiopulmonary resuscitation (CPR) should be performed immediately. Any child who becomes unconscious before, during or after rescue should be taken to a trained medical professional to assess and treat for medical complications that may negatively impact health outcomes.^4^

The Sundarbans region of West Bengal, India is a rural, deltaic region with high rates of poverty. This region experiences one of the highest recorded rates of child drowning globally.^5^ The Sundarbans suffers from poor health system reach, inadequate connectivity between towns and villages and low rates of literacy, making fast response to drowning events difficult.^6^ Our previous study into child drowning events in the Sundarbans region revealed that only 16.6% of child drowning victims were taken to a healthcare practitioner, most of whom were local doctors with limited formal qualifications.^5^

Currently, we have a limited understanding of the care pathway sought and received postfatal and non-fatal drowning events. This understanding is important for context-specific prioritisation of drowning prevention interventions and the design of interventions that consider community realities.

The three-delay framework describes three delays that may increase mortality due to critical conditions, such as childbirth complications: (1) deciding to seek medical care, (2) reaching an appropriate medical care facility and (3) receiving suitable and timely treatment at the facility.^7 8^

Applications of the three-delay framework in other low-income contexts have found that for various health conditions, the first delay may be influenced by factors such as perception of injury severity, perceptions of cost and quality of treatment, and socioeconomic status. The second delay may be caused by modes of transport available, transport costs and quality of roads. The third delay may be influenced by a lack of resources at the hospital, a lack of knowledge of medical practitioners and non-adherence caused by religious or cultural beliefs.^7 9 10^

We hypothesise that many of these delay factors will be present in the Sundarbans context but may differ in their presentation due to unique challenges faced by communities in this remote and socioeconomically disadvantaged region. Additionally, we hypothesise that for often poorly identifiable incidents like child drowning, there may be another delay before delay one—a delay in detecting that the drowning event has occurred. Faster rescue time is instrumental in ensuring higher survival rates,^4^ and yet limited knowledge of appropriate rescue and resuscitation techniques, limiting cultural beliefs, poor supervision and unclear water in water bodies in low-income contexts may exacerbate this delay.^11 12^ This study will apply the three-delay framework to drowning for the first time in our knowledge to assess for this additional delay.

The patient journey mapping method is a tool used to identify an improved patient journey for a postevent victim. Victims’ experiences postevent are carefully followed and recorded, often through a descriptive qualitative content analysis approach.^13^ This method can be applied to drowning events in the Sundarbans context to identify the delays occurring postevent and the factors that may be contributing to those delays.

## Materials and methods

### Aims

This study aims to identify the postdrowning event patient care journey experienced by children in the Sundarbans and the delays preventing access to effective care, using the three-delay framework.

### Data collection

Data were collected by a trained male data collector bilingual in Bengali and English and contracted full-time as part of a larger project in the Sundarbans to assess drowning burden and risks. To address potential discomfort caused by gender or educational differences between the data collector and participants, village leaders or community health workers already engaged in the larger project facilitated introductions. The data collector held a Master’s degree in anthropology and had 3 years of field experience in qualitative data collection.

Households of child drowning victims of both fatal and non-fatal outcome drownings in the last 1 year were purposively selected and interviewed to understand the postevent journey. Cases were sought from eight rural Gram Panchayats (the smallest administrative unit in India) with a high drowning burden and diverse contexts with respect to rurality, type of water bodies and access to health services.^5 14^ Cases were identified by the introduction of community leaders in these Gram Panchayats. Data collection occurred between 1 October 2024 and 5 February 2025.

Interviews were held at the home of the participant or another private area that was comfortable to the participant. Only the interviewer and participant were present. Interviews were audio recorded. Interviewers encouraged the participants to share their experience in the form of storytelling, with relevant probes (see online supplemental file S1). These incorporated different stages of the journey, including rescue, resuscitation, transport to care facilities or health providers, types of care received in facilities or by health providers and postdischarge care. At each step, the actors involved and decisions made were noted.

### Participants

Adults over the age of 18 years were interviewed. The participants were required to be present before, during and after the drowning event. Where there was no one adult present throughout the entire post-event journey and available to participate, additional participants were sought through a snowball method to record missing components of the postevent journey. Participants included victims’ parents, grandparents, older siblings, neighbours and local doctors.

### Patient and public involvement

Communities and local organisations involved in the larger Indian Council of Medical Research project, of which this study is a part, were involved in identifying research questions. Community leaders were also engaged in identifying and briefing participants. Community leaders and partner organisations are also involved in the dissemination of the broader findings of the project with communities.

### Sample size

Cases of fatal and non-fatal outcome drownings from the previous 1 year were identified. The cases were required to be of children aged 0–15 years. Sample size could not be determined a priori; however, based on the previous survey data and a limited sample frame of eight Gram Panchayats, we expected to develop 16–20 maps. Data collection was completed when data saturation was reached, once new themes were not emerging from the interviews.

### Analysis

All Bengali transcripts were transcribed and translated into English for analysis. Interviews were not repeated. Transcripts were not returned to participants for comment due to the logistical and literacy barriers. Transcripts were saved on a password-protected and secured institutional folder.

The delays in accessing appropriate care were mapped against the three-delay framework and a fourth hypothesised delay in Excel by MG, namely: (0) delay at the time of rescue, (1) delays in deciding to seek medical care, (2) barriers in reaching an appropriate medical care facility and (3) whether the victim received suitable and timely treatment at the facility. Mapping was verified by JJ.

## Results

A total of 18 case studies were collected, the details of which were explored through 37 interviews of adults present during and after the event. No participants approached refused to participate (see online supplemental file S2 for details of all cases). Where information was missing from a case study, such as the first participant only witnessing part of the postdrowning journey, other witnesses who were present for the remainder of the journey were identified and interviewed. The interviews ran between 10 and 30 min.

The cases were of children aged between 1 and 15 years old, with a mix of male and female victims. Most of the events occurred during the daytime, before 14:00 hours, though some cases occurred later in the afternoon and at night. In all the cases, the child fell into a pond or river near their own home or a relative’s home.

**Figure 1:**
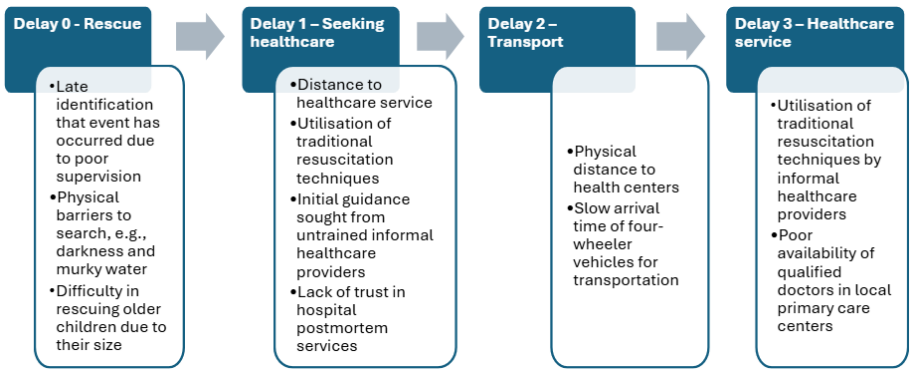
Factors leading to delays postdrowning event.

The results are described below as per the delays of the three-delay model and summarised in figure 1. We found that for injuries like drowning, there existed an instrumental ‘delay zero’, a delay at the time of rescue. The results are described under each of the delays below.

### Delay 0: recognising the drowning event and rescuing the child

The time taken to identify that the child had fallen into water and was drowning was between 30 s to more than 30 min. When caretakers, most often parents or grandparents, were within view of the water body, they were faster to identify the drowning event as they were more likely to notice the child was missing. However, if the caretakers were out of sight or earshot, they often did not begin searching until later. In many cases where caretakers were nearby, they reported hearing no sounds, suggesting that visual attention is imperative for awareness around drowning.

In two cases, children were playing with other children when they fell, and their companions alerted adults. This caused a delay while the children searched for and found a nearby adult.

He (brother of victim) is calling, “Mom, come quickly!” He is calling by her name that (the victim) has fallen into the water. We were not paying much attention as we were busy in our work. – Parent, Case 3

In all cases, children were rescued by members of the nearby community, such as family or neighbours. Rescue was never conducted by someone with training or practice in safe water rescue.

It’s essential that the rescue time is quick…. But in many cases the parents of the child are careless. If there is a delay, there is a danger of losing life in the water. Then there is nothing we can do. – Local informal doctor, Case 18

A key barrier to fast rescue was the nature of water bodies, which were often filled with vegetation, increasing the difficulty of locating the child. In a few cases, rescuers used nets to find the child in the water.

Some cases also occurred during the rainy monsoon season, when the water bodies were larger and deeper. This also delayed rescue efforts as only rescuers who were strong in swimming could effectively enter the water, and in some cases, it took time to find someone who could reach the child.

In cases where the child was larger and older, rescue also took more time as it was more difficult to pull the child out. No objects were used to aid the rescue of older children, such as floating bottles or rope, and rescuers relied on their own swimming abilities.

It took 5-7 minutes to pick him from the water… at 15 years old he was quite heavy… we pulled with his clothes. – Neighbour, Case 17

Some of the drowning events also occurred at night, often while parents were busy in meal preparation and children were playing near the home outside. The communal nature of rural living meant that children frequently played with neighbouring children outside even after daylight hours. The nighttime darkness increased the difficulty of the search. Rescuers required time to find torches and search nearby areas and water bodies.

Then we searched along the roads and then returned…a boy standing beside a house on the other side of road was asking his mother for a torch to see something floating in the pond. After that, when she came with the torch, they found the boy floating in the middle of the pond. – Neighbor, Case 1

### Delay 1: deciding to seek medical care

In the majority of cases, neighbours would hear the commotion of the search and rescue and were present at the time the child was pulled from the water. Hence, decision-making about the child’s medical care was usually made by a group of people at the scene of the event.

The most common response postincident was to press the child’s stomach or to spin the child upside down over the head. In some cases, children were also given holy water from a nearby temple or massaged with oil. The delay in seeking medical care occurred while rescuers attempted these responses, as a decision to seek medical care was usually taken after these attempts were not effective.

…My father and uncles all used to say that when a child falls into the water, we should spin them upside down. That’s why I did that. – Father of victim, Case 11

Rescuers also attempted these responses first because medical care facilities were situated far from the event site, so they were concerned the child would not arrive in time to save them. In some cases, while a primary health centre was available nearby, the centre was known to be poorly manned, with the doctors’ schedule uncertain.

Participant: There is one primary health centre here in the market but no one knows when the doctor comes there.

Interviewer: If something happens to any of the people here at night, then they come here?

A: Yes, there is no one else in this village. – Local informal doctor, Case 16

Children who were unconscious or showed signs of great distress, such as coughing and crying, were most often taken for medical care, as onlookers believed it gave the only chance of saving the child. In most cases where the child was conscious after rescue, medical treatment was not sought.

In one case where the child was already considered dead, the onlookers did not take the child to any medical facility as they did not see any requirement. In two other cases where the child was considered dead, the family did not wish to take their child for a postmortem as they did not trust the hospital to release the body on time, which would delay religious death rituals.

We are afraid of doing a postmortem in case we don’t get to complete our rituals… so we are forbidden by our religion. Parent, Case 16.

Only in one case was a car called immediately to take the child to the hospital, when the onlookers believed formal medical care was required, as traditional techniques were unlikely to work.

Seeing that the child’s condition was bad, I said, “First take her to the hospital.” The thought that I should move her, shake her didn’t come to my mind because I saw that the child’s condition was very bad. – Neighbour, Case 10.

### Delay 2: reaching an appropriate medical care facility

In most cases, local informal doctors were involved, who were unlicensed practitioners applying traditional healing knowledge. Local informal doctors arrived at the event site as they lived in the village and were brought by onlookers, or they themselves heard the rescue occur. In three cases, the informal doctors were family members of the victim and were easily accessible.

Because there is only one, there is no other doctor other than that [local informal doctor]. Everyone goes to him.- Neighbour, Case 11

Some of the informal doctors recommended that the children be taken to the hospital as they believed they could do nothing, but these children did not survive after reaching.

Due to the wait time required to find and call a rental car or e-rickshaw, children were usually transported by a bike owned by the family or a neighbour. In cases where the child was older and would be difficult to transport on a bike, they had to wait for a larger vehicle.

Hospitals were up to 30 km away from the event sites. Participants’ families noted that the distance may have led to untimely treatment.

We have nothing here except the primary block hospital, which is 25 to 30 kilometres away… If something happens, it takes about 15 to 20 minutes to find a car. – Parent, Case 13

### Delay 3: receiving suitable and timely treatment

Except in one case, local informal doctors who attended to the victims did not use appropriate resuscitation techniques like CPR. They pressed the victims’ stomachs or spun them to remove water from the child’s body. Three of the informal doctors were unable to revive the child, so they recommended that the children be taken to a formal medical facility. In one case where the village was closer to the major city of Kolkata, the local informal doctor administered CPR resuscitation techniques as he had attended a training workshop in the city recently.

Participant: I gave [CPR]. I've saved four boys before.

Interviewer: Did you save four?

Participant: I have saved four. I thought about this CPR training. - Local informal doctor, Case 12

In three cases, registered doctors were available nearby and attended the scene. These doctors employed CPR techniques.

In most cases when children were taken to the hospital, they were immediately seen to. However, in two of these cases, parents noted that this fast response from the hospital staff was unusual, as emergency care at hospitals is typically delayed. Respondents commented that care at the hospitals appeared adequate once received.

Four of the children who were taken to the hospital were pronounced dead, attributed to a delay in rescue or incorrect resuscitation. Other children taken to the hospital survived after examination and provision of oxygen.

## Discussion

This study aimed to identify key delays that occurred in the postdrowning medical care journey of children in the Sundarbans region of India. This low-income region faces many challenges to a fast and effective response. The cases identified considered a number of regions across the Sundarbans, including those closer and further to health centres, which provided a range of perspectives on delays faced postdrowning.

As per the three-delay framework, we found that not only were delays occurring as per the phases in the model, but a crucial delay was also occurring at the time of identification of the event and rescue, which we dubbed delay zero. Delay zero was attributed to inadequate adult supervision, physical and environmental barriers to rescue, unavailability of resources to aid rescue and use of inappropriate rescue techniques.

Rescue time is a vital predictor of survivability, with submersion time less than 6 min associated with a significantly higher chance of survival with minimal cognitive or physical defect.^1 15^ However, fast rescue was marred by multiple factors, such as a lack of supervision and low visibility from murky water and night-time darkness. Unfortunately, these factors are difficult to mitigate. A key intervention evaluated to improve supervision is the provision of supervised childcare during caretakers’ busiest times, between 10:00 and 14:00 hours, where children can be carefully monitored.^3 16^ There are also preliminary findings that suggest fencing ponds may be used to prevent young children from entering water bodies, though further research on the sustainability and effectiveness of such interventions is required.^17 18^ Our results demonstrate the importance of implementing preventative measures, as once drowning events occur in these contexts, timely rescue is difficult.

Another concern identified in the results was that larger children were difficult to rescue efficiently and required strong swimmers to remove them from the water. International rescue standards recommend that larger children and adults be rescued from dry land with the assistance of objects such as poles, rope and flotation devices so as not to put the rescuer at risk.^19^ The lack of knowledge and availability of these objects means that rescue is delayed, and additional risk is created for the rescuer. Again, community sensitisation and ensuring all home ponds have locally sourced flotation devices available may assist in drowning event rescues. In other LMIC contexts, affordable flotation devices have been made from large plastic bottles tied to rope.^20^

Immediate and prolonged administration of appropriate CPR is also associated with improved survival outcomes.^1^ Delay one, the decision to seek medical treatment, was often caused by bystanders applying incorrect methods of resuscitation, such as pressing the child’s stomach. This behaviour was reinforced as most informal local doctors, who are trusted by the community, administered the same techniques. Community sensitisation is essential to encourage people to immediately seek individuals who can administer CPR on the victim if they are unconscious. Strengthening primary healthcare systems by ensuring availability of licensed and trained doctors in rural areas is also essential. Availability of such individuals in communities can be increased through training adults and local informal doctors in CPR techniques and informing communities on whom to call when an event occurs.^3^

The drowning chain of survival identifies five steps to improve drowning survival rates: prevent drowning, recognise distress, provide flotation, remove from water and provide care as needed.^21^ However, our results show significant challenges in each of these steps due to poor rescuer and resuscitator knowledge, geographical challenges and deficiencies in the reach of health systems. Overall, our data did not show major delays with treatment once victims reached formal medical care settings like hospitals. However, the non-availability of registered doctors and medical care facilities near communities spoke to the broader inaccessibility of the healthcare system in the Sundarbans region. Even where primary health centres were built, they were not adequately staffed and followed inconsistent schedules. According to the latest data released by the West Bengal state in 2018, the districts of North 24 Parganas and South 24 Parganas, across which the Sundarbans region is spread, only housed 88 health clinics with a bed ratio of 0.07 per 1000 population.^22^ The Indian National Health Policy 2017 recommends 2 beds per 1000 population.^23^ For drowning victims to receive timely care, the health system reach must be improved both in terms of geographical access and staff availability. This can be improved by localised interventions such as the provision of scheduled and predictable mobile boat health clinics and improved staffing at primary health centres.^24^

Reframing the three-delay model as the four-delay model, adding delay zero at rescue, allows the drowning prevention community to focus on the difficulty of timely rescue in this low-income setting and the importance of drowning prevention interventions that reduce the occurrence of drowning in the first place. Strengthening the healthcare system and training local residents and informal doctors may improve after-event care and outcomes, but these will have limited impact if children are not being rescued in a timely manner.

Our research emphasised the importance of focusing resources and attention on preventative measures in this challenging context, as well as appropriate rescue and care response. These may involve the introduction of physical barriers around water bodies, the creation of safe, supervised spaces for children and providing swimming and CPR training to older children and adults. Communities can be empowered to take ownership of such interventions. However, system-level support from governments is also required to improve the reach of health systems to rural locations. Future research may consider applying this updated framework to other injuries or health events that are recognised with some delay, as this may provide opportunities for faster and more effective care in a range of conditions.

### Limitations

The current study identified 18 child drowning cases across eight Gram Panchayats in the Sundarbans. The geographical coverage of the study may limit the generalisability of the findings to other low-income contexts, and similar studies in other high-risk regions may be required to identify context-specific delays occurring elsewhere. Another limitation is that all data were collected retrospectively, and although we mapped recent events, recall bias may be present. A future study may seek to follow real-time drowning events occurring in high-risk communities to capture decision-making processes through observation. Finally, the collection of income data may have aided in the impact of socio-economic conditions on delays, highlighting nuances within vulnerable groups.

## Conclusions

Drowning is a major cause of child mortality and morbidity in the Sundarbans and is exacerbated by poor postdrowning care and response. Drowning, in particular, suffers from a unique delay in postevent care, delay zero, where rescue is not occurring in a timely manner. This delay is caused by factors that are difficult to mitigate in a low-income context, such as poor supervision and physical characteristics of the water. Hence, prevention is key. However, other delays can be improved by training local informal doctors and bystanders in appropriate resuscitation techniques and sensitising communities to fast rescue and medical help-seeking.

## Supplementary material

10.1136/bmjopen-2025-103099 online supplemental file 1

## Data Availability

Data are available on reasonable request.
