# Supplementary material for: Drowning in delays: a patient journey mapping analysis of child drowning care in rural India
Source: BMJ Open. 2025 Aug 24;15(8):e103099. doi: 10.1136/bmjopen-2025-103099 (PMC12382531; doi:10.1136/bmjopen-2025-103099)
Supplement: online supplemental file 1 [file bmjopen-15-8-s001.docx]

**Supplementary File S1 - Probes**

Please tell us everything you recall from just before the drowning incident to the finalization or recovery of all health care services sought.

Probing questions:

1. Who rescued the child?
2. How did the rescuer know that the child had fallen?
3. How long did rescue take? Why did it take so long?
4. Who was present when the rescue happened?
5. What did you do with the child after taking them from the water?
6. Did you take the child to the doctor? Which doctor? Why or why not?
7. How long did reaching the doctor/hospital take?
8. What care was given by the doctor/hospital?
9. What happened to the child after discharge?

**Supplementary File S2 – List of cases**

| **Case number** | **Age of victim** | **Gender of victim** | **Fatal/non-Fatal** | **No. Of Interviews** |
| --- | --- | --- | --- | --- |
| 1 | Early childhood (2-5 years) | Male | Non-fatal | 1 |
| 2 | Early childhood (2-5 years) | Male | Non-fatal | 1 |
| 3 | Early adolescence (12 - 17 years) | Male | Non-fatal | 1 |
| 4 | Middle childhood (6 - 11 years) | Female | Non-fatal | 3 |
| 5 | Toddler (13 months - 2 years) | Female | Fatal | 2 |
| 6 | Early childhood (2-5 years) | Female | Fatal | 2 |
| 7 | Early childhood (2-5 years) | Female | Fatal | 2 |
| 8 | Early childhood (2-5 years) | Female | Non-fatal | 1 |
| 9 | Early childhood (2-5 years) | Male | Non-fatal | 1 |
| 10 | Middle childhood (6 - 11 years) | Male | Fatal | 3 |
| 11 | Toddler (13 months - 2 years) | Male | Fatal | 3 |
| 12 | Early adolescence (12 - 17 years) | Male | Non-fatal | 3 |
| 13 | Early childhood (2-5 years) | Male | Non-fatal | 2 |
| 14 | Early childhood (2-5 years) | Female | Non-fatal | 2 |
| 15 | Early childhood (2-5 years) | Male | Fatal | 2 |
| 16 | Early childhood (2-5 years) | Male | Fatal | 3 |
| 17 | Early adolescence (12 - 17 years) | Male | Fatal | 2 |
| 18 | Early childhood (2-5 years) | Female | Non-fatal | 3 |

infancy (28 days - 12 months), toddler (13 months - 2 years), early childhood (2 - 5 years), middle childhood (6 - 11 years), early adolescence (12 - 17 years)
