# Supplementary material for: Drowning in delays: a patient journey mapping analysis of child drowning care in rural India
Source: BMJ Open. 2025 Aug 24;15(8):e103099. doi: 10.1136/bmjopen-2025-103099 (PMC12382531; doi:10.1136/bmjopen-2025-103099)
Supplement: Supplementary file 2 [file bmjopen-2025-103099.reviewer_comments.pdf]

PEER REVIEW HISTORY

BMJ Open publishes all reviews undertaken for accepted manuscripts. Reviewers are asked to complete a checklist review form and are provided with free text boxes to elaborate on their assessment. These free text comments are reproduced below.

ARTICLE DETAILS

Title (Provisional)

What causes delays in post-drowning care of children in low-income settings? A qualitative patient journey mapping analysis in rural India

Authors

Jagnoor, Jagnoor; Gupta, Medhavi

VERSION 1 - REVIEW

**Reviewer**	**1**
**Name**	**Beerman, Stephen**
**Affiliation**	**University of British Columbia Clinical Associate Professor and Department of Family Practice, Site Director., Dept of family practice**
**Date**	**04-Jun-2025**
**COI**	**None**

Thank you for your work on this very helpful scholarship that is in a setting of great interest and a gap issue that needs attention

Abstract:

Objectives: Consider using regular flooding area vs "waterlogged"

Strengths and Limitations: In the 5th/last point listed consider removing the word "initially" as I do not think this social information was included initially or later.

Introduction:

Page 4 line ll consider a review of the word "inadequate" - a softer and less judgemental term for consideration could be immature or less developed or below expectations or wanting --- in low income context.

Page 4 line 15 consider improvements to the tne of this sentence. Currently this says - ---- "and treat for internal issues such as residual fluid in the lungs" Consider stating a more generic phrase such as -----and assess and treat for post drowning complications that can negatively impact outcomes.

Page 4 Line 32 - consider replacing the word ground in the phrase "consider ground realities" to community - ie consider community realities.

Page 4 Line 53/54 - consider changing the word "underdeveloped" to a lower socioeconomic region.

Page 4 Line 58 - consider improving the phrase - "poor community knowledge" such as low water safety evidence based prevention and CPR knowledge -------

Materials and Methods:

Data collection: Consider informing readers if the data collector was multi-lingual.

Page 6 Line 35 - consider adding the word outcomes as in fatal and non-fatal drowning outcome in the last one year-----

Page 6 Line 43 - consider using more unambiguous date documentation. Jan 10th 2024 to May 2nd 2025 or Oct 1st 2024 to Feb 5th 2025.

Participants:

Page 7 Line 6 - consider adding the word "be" ie to be present before, during and after----

Page 7 Line 7 - clean up the language here. maybe a simple as remove the word "one"

Results:

5.1 Delay 0 to 5.4 Delay 3

Page 8 Line 51 consider changing "muddied and filled with bracken" with something like - low visibility and vegetation filled water bodies.

As I read through the Delay 0 section (5.1) I not that the prevention gaps are:

a) Adult direct supervision or in vision observation

b) Barriers

c) Rescue aids (reaching, floating, paddling/rowing)

d) Awareness of increased risk from night bathing or night water play

e) Evidence Based CPR training

I have an appetite to see this in the discussion in a more strongly worded manner. You point out that in low resource settings drowning is more likely to be fatal due to gaps in rescue, awareness and medical care. I am aware that this study is focused on using this method to illuminate the gaps. It is also an opportunity to provide guidance on gap reduction/life saving interventions.

Discussion - consider adding after Page 11 Line 56/57 a more detailed and clear improvement plan proposal based on the data above.

References, figure and supplimental files are very helpful - thank you

**Reviewer**	**2**
**Name**	**Cenderadewi, Muthia**
**Affiliation**	**James Cook University**
**Date**	**08-Jul-2025**
**COI**	**None**

General comment:

Thank you for the opportunity to review this manuscript. Overall, it is a well-written and timely contribution that addresses a critical gap in the drowning prevention literature, particularly concerning post-event responses to child drowning in resource-limited settings. I offer the following suggestions for the authors' consideration.

Specific comments:

• Page 4, lines 4–5: The statement that "this study will apply the three-delay framework to drowning for the first time..." may come across as overly definitive. Consider rephrasing with a qualifier such as "to our knowledge" to maintain academic caution.

• Page 4, lines 22–25: The sentence "This study aims to apply the three-delay framework and identify the post-drowning event patient care journey experienced by children in the Sundarbans, identifying the delays faced preventing access to effective care" could benefit from reordering for clarity. Consider revising to: "This study aims to identify the post-drowning event patient care journey experienced by children in the Sundarbans and the delays preventing access to effective care, using the three-delay framework."

• Page 4, lines 29–30: The sentence notes that data were collected by a trained male data collector. It may be valuable for the authors to reflect on whether the gender of the interviewer influenced the depth or nuance of responses, particularly given the cultural context of the Sundarbans. For instance, did mothers or female caregivers feel comfortable sharing detailed accounts of the post-drowning care experience with a male interviewer? Might the perceived social distance between participants and a highly educated male have shaped the nature or depth of their responses? A brief discussion of these potential influences and any strategies used to address them would enhance the methodological transparency and reflexivity of the study.

• Page 4, lines 35–36: The manuscript states that "families of child drowning victims of both fatal and non-fatal drownings in the last one year were purposively selected and interviewed..." It would be helpful to clarify what is meant by "families" in this context. Were interviews conducted primarily with parents, or did they also include grandparents, siblings,

or other caregivers? Do readers need to appraise how representative they are of the broader caregiving context?

• Page 8, lines 12–16: The manuscript notes that some drowning events occurred at night, often while parents were engaged in meal preparation, and that darkness complicated rescue efforts. Did the participants discuss any sociocultural or contextual factors contributing to children being unsupervised near water during evening hours, for example evening play routines, bathing practices, or environmental hazards such as lack of designated safe pathways between homes and water sources? Including such insights could deepen the understanding of contextual vulnerabilities and inform more targeted prevention strategies.

• Page 8, line 26: The phrase “local informal doctors” requires clarification. The term “doctor” typically implies formal medical training and certification, so it would be helpful to specify who is being referred to here. Does this term refer to unlicensed practitioners, village health workers, pharmacy-based providers, or possibly interns or doctors in training?

• Page 9, lines 17–18: The statement “However, in two of these cases, parents noted that this fast response was unusual” is somewhat ambiguous and would benefit from further clarification. Does this imply that emergency response is typically delayed, and that these cases were exceptions? It would also be valuable to know whether participants commented not only on the timeliness but also on the quality of medical services received, such as perceived competence of providers, appropriateness of treatment, or adequacy of facilities. Including such reflections would offer a more comprehensive understanding of post-drowning care experiences.

• Page 10, lines 33–34: The statement “However, our results show significant challenges in each of these steps due to poor rescuer and resuscitator knowledge, geographic challenges and health-system deficiencies” would benefit from greater specificity. Which findings specifically demonstrate health-system deficiencies? Was this referring to a lack of trained personnel and inadequate medical facilities, or something else? As I understand it, the study did not report delays in treatment once families reached health facilities, so it would be helpful to clarify what constitutes the cited health-system limitations.

VERSION 1 - AUTHOR RESPONSE

Reviewer 1

Thank you for your work on this very helpful scholarship that is in a setting of great interest and a gap issue that needs attention

Response: Thank you for your comment.

Objectives: Consider using regular flooding area vs "waterlogged"

Response: The abstract is updated to:

"Setting: The study was conducted in the Sundarbans, a rural region in India facing a high burden of child drowning due to high presence of open water and regular flooding."

Strengths and Limitations: In the 5th/last point listed consider removing the word "initially" as I do not think this social information was included initially or later.

Response: This has been removed

Introduction:

Page 4 line ll consider a review of the word "inadequate" - a softer and less judgemental term for consideration could be immature or less developed or below expectations or wanting --- in low income context.

Response: This has been updated to “wanting”.

Page 4 line 15 consider improvements to the tone of this sentence. Currently this says - ----"and treat for internal issues such as residual fluid in the lungs" Consider stating a more generic phrase such as -----and assess and treat for post drowning complications that can negatively impact outcomes.

Response: This has been updated to: “…to assess and treat for medical complications that may negatively impact health outcomes.”

Page 4 Line 32 - consider replacing the word ground in the phrase "consider ground realities" to community - ie consider community realities.

Response: This has been updated as suggested.

Page 4 Line 53/54 - consider changing the word "underdeveloped" to a lower socioeconomic region.

Response: This has been updated to “socio-economically disadvantaged region”.

Page 4 Line 58 - consider improving the phrase - "poor community knowledge" such as low water safety evidence based prevention and CPR knowledge -------

Response: This has been updated to "…limited knowledge of appropriate rescue and resuscitation techniques…"

Materials and Methods:

Data collection: Consider informing readers if the data collector was multi-lingual.

Response: This has been updated.

Page 6 Line 35 - consider adding the word outcomes as in fatal and non-fatal drowning outcome in the last one year-----

Response: This has been updated as suggested.

Page 6 Line 43 - consider using more unambiguous date documentation. Jan 10th 2024 to May 2nd 2025 or Oct 1st 2024 to Feb 5th 2025.

Response: This has been updated

Participants:

Page 7 Line 6 - consider adding the word "be" ie to be present before, during and after----

Response: This has been added

Page 7 Line 7 - clean up the language here. maybe a simple as remove the word "one"

Response: This has been removed

Results:

5.1 Delay 0 to 5.4 Delay 3

Page 8 Line 51 consider changing "muddied and filled with bracken" with something like - low visibility and vegetation filled water bodies.

Response: This has been updated as suggested

As I read through the Delay 0 section (5.1) I note that the prevention gaps are:

a) Adult direct supervision or in vision observation

b) Barriers

c) Rescue aids (reaching, floating, paddling/rowing)

d) Awareness of increased risk from night bathing or night water play

e) Evidence Based CPR training

I have an appetite to see this in the discussion in a more strongly worded manner.

Response: Thanks for the suggestion. We added a summarising sentence in the discussion as follows: “Delay zero was attributed to inadequate adult supervision, physical and environmental barriers to rescue, unavailability of resources to aid rescue and use of inappropriate rescue techniques.”

You point out that in low resource settings drowning is more likely to be fatal due to gaps in rescue, awareness and medical care. I am aware that this study is focused on using this method to illuminate the gaps. It is also an opportunity to provide guidance on gap reduction/life saving interventions. Discussion - consider adding after Page 11 Line 56/57 a more detailed and clear improvement plan proposal based on the data above.

Response: Keeping in mind the word count, the following has been added to the final paragraph of the discussion:

“Our research emphasised the importance of focussing resources and attention on preventative measures in this challenging context, as well as appropriate rescue and care response. These may involve the introduction of physical barriers around water bodies, creation of safe, supervised spaces for children, and providing swimming and CPR training to older children and adults. Communities can be empowered to take ownership of such interventions. However, system-level support from governments is also required to improve reach of health systems to rural locations.”

Reviewer: 2

General comment:

Thank you for the opportunity to review this manuscript. Overall, it is a well-written and timely contribution that addresses a critical gap in the drowning prevention literature, particularly concerning post-event responses to child drowning in resource-limited settings.

Response: Thank you for your positive comment.

Specific comments:

• Page 4, lines 4–5: The statement that "this study will apply the three-delay framework to drowning for the first time..." may come across as overly definitive. Consider rephrasing with a qualifier such as "to our knowledge" to maintain academic caution.

Response: This has been added to the sentence as suggested.

• Page 4, lines 22–25: The sentence “This study aims to apply the three-delay framework and identify the post-drowning event patient care journey experienced by children in the Sundarbans, identifying the delays faced preventing access to effective care” could benefit from reordering for clarity. Consider revising to: “This study aims to identify the post-drowning event patient care journey experienced by children in the Sundarbans and the delays preventing access to effective care, using the three-delay framework.”

Response: This has been revised as suggested.

• Page 4, lines 29–30: The sentence notes that data were collected by a trained male data collector. It may be valuable for the authors to reflect on whether the gender of the interviewer influenced the depth or nuance of responses, particularly given the cultural context of the Sundarbans. For instance, did mothers or female caregivers feel comfortable sharing detailed accounts of the post-drowning care experience with a male interviewer? Might the perceived social distance between participants and a highly educated male have shaped the nature or depth of their responses? A brief discussion of these potential influences and any strategies used to address them would enhance the methodological transparency and reflexivity of the study.

Response: We did not collect feedback on the impact that data collector gender/education had on responses of participants. However, we were aware of this potential issue, and did follow practices to ensure comfort of participants. We have added the following sentence:

“To address potential discomfort caused by gender or educational differences between the data collector and participants, village leaders or community health workers already engaged in the larger project facilitated introductions.”

• Page 4, lines 35–36: The manuscript states that “families of child drowning victims of both fatal and non-fatal drownings in the last one year were purposively selected and interviewed...” It would be helpful to clarify what is meant by “families” in this context. Were interviews conducted primarily with parents, or did they also include grandparents, siblings, or other caregivers? Do readers need to appraise how representative they are of the broader caregiving context?

Response: We updated the word Families to “Households” to better reflect the selection process. As mentioned in section 4.3, participants were any adult present during and/or after the drowning event. We have also added the following sentence: “Participants included victims’ parents, grandparents, older siblings, neighbors and local doctors.”

• Page 8, lines 12–16: The manuscript notes that some drowning events occurred at night, often while parents were engaged in meal preparation, and that darkness complicated rescue efforts. Did the participants discuss any sociocultural or contextual factors contributing to children being unsupervised near water during evening hours, for example evening play routines, bathing practices, or environmental hazards such as lack of designated safe pathways between homes and water sources? Including such insights could deepen the understanding of contextual vulnerabilities and inform more targeted prevention strategies.

Response: We have added the following to the results on page 7:

“Some of the drowning events also occurred at night, often while parents were busy in meal preparation and children were playing near the home outside. The communal nature of rural living meant that children frequently played with neighbouring children outside even after daylight hours.”

• Page 8, line 26: The phrase “local informal doctors” requires clarification. The term “doctor” typically implies formal medical training and certification, so it would be helpful to specify who is being referred to here. Does this term refer to unlicensed practitioners, village health workers, pharmacy-based providers, or possibly interns or doctors in training?

Response: We have added the clarification – ‘In most cases, local informal doctors were involved, who were unlicensed practitioners applying traditional healing knowledge.”

• Page 9, lines 17–18: The statement “However, in two of these cases, parents noted that this fast response was unusual” is somewhat ambiguous and would benefit from further clarification. Does this imply that emergency response is typically delayed, and that these cases were exceptions? It would also be valuable to know whether participants commented not only on the timeliness but also on the quality of medical services received, such as perceived competence of providers, appropriateness of treatment, or adequacy of facilities. Including such reflections would offer a more comprehensive understanding of post-drowning care experiences.

Response: The following details have been added:

"In most cases where children were taken to the hospital, they were immediately seen to. However, in two of these cases, parents noted that this fast response from the hospital staff was unusual, as emergency care at hospitals is typically delayed. Respondents commented that care at the hospitals appeared adequate once received."

• Page 10, lines 33–34: The statement "However, our results show significant challenges in each of these steps due to poor rescuer and resuscitator knowledge, geographic challenges and health-system deficiencies" would benefit from greater specificity. Which findings specifically demonstrate health-system deficiencies? Was this referring to a lack of trained personnel and inadequate medical facilities, or something else? As I understand it, the study did not report delays in treatment once families reached health facilities, so it would be helpful to clarify what constitutes the cited health-system limitations.

Response: This is referring to poor reach of health systems in rural regions, rather than health-system issues at the tertiary level. We have updated this sentence to say: "However, our results show significant challenges in each of these steps due to poor rescuer and resuscitator knowledge, geographic challenges and deficiencies in the reach of health systems."

We have also added this sentence discussing first responder training requirements: "Strengthening of primary healthcare systems by ensuring availability of licenced and trained doctors in rural areas is also essential."

VERSION 2 - REVIEW

**Reviewer**	**2**
**Name**	**Cenderadewi, Muthia**
**Affiliation**	**James Cook University**
**Date**	**07-Aug-2025**
**COI**	

Thank you for the opportunity to review the revised version of this manuscript. The authors have made appropriate amendments in response to previous feedback. The paper is relevant to the journal's scope and offers meaningful contributions to the field, particularly in the context of drowning prevention in LMICs.
